# Diabetes alters cardiorespiratory dynamics: insights from short-term recurrence quantification analysis of pulse-respiration quotient

**DOI:** 10.3389/fphys.2025.1584922

**Published:** 2025-04-25

**Authors:** José Javier Reyes-Lagos, Kioko Guzmán-Ramos, Joel Lomelí, Adriana Cristina Pliego-Carrillo, Miguel Ángel Peña-Castillo, Pedro López-Sánchez, Virgilio Eduardo Trujillo-Condes, Laura Ivoone Garay-Jiménez, Juan Carlos Echeverría, María Fernanda Villegas-Zarco, Laura Mercedes Santiago-Fuentes

**Affiliations:** ^1^ Facultad de Medicina, Universidad Autónoma del Estado de México, Toluca de Lerdo, Toluca, Mexico; ^2^ Departamento de Ciencias de la Salud, Universidad Autónoma Metropolitana Unidad Lerma, Lerma, Mexico; ^3^ Escuela Superior de Medicina, Instituto Politécnico Nacional, Miguel Hidalgo, Mexico City, Mexico; ^4^ Departamento de Ingeniería Eléctrica, Universidad Autónoma Metropolitana Unidad Iztapalapa, Iztapalapa, Mexico City, Mexico; ^5^ Unidad Profesional Interdisciplinaria en Ingeniería y Tecnologías Avanzadas, Instituto Politécnico Nacional, Mexico City, Mexico; ^6^ Departamento de Ciencias de la Salud, Universidad Autónoma Metropolitana Unidad Iztapalapa, Iztapalapa, Mexico City, Mexico

**Keywords:** pulse-respiration quotient, type 2 diabetes mellitus, cardiorespiratory coupling, recurrence quantification analysis, nonlinear dynamics

## Abstract

**Introduction:**

The Pulse-Respiration Quotient (PRQ) is considered a powerful tool for assessing dynamic interactions between cardiac and respiratory rhythms. Type 2 diabetes mellitus (T2DM) disrupts autonomic control, potentially compromising the complexity and adaptability of cardiorespiratory dynamics. In this cross-sectional, exploratory study, we investigated whether T2DM alters cardiorespiratory dynamics by analyzing short-term PRQ signals using conventional linear indices and Recurrence Quantification Analysis (RQA).

**Methods:**

Thirty-eight participants (20 T2DM and 18 controls) completed four standardized tasks—supine rest, orthostatic challenge, paced breathing, and the Valsalva maneuver—while electrocardiographic and respiratory signals were continuously recorded. From these signals, R-to-R peak interval (RRI) and breath-to-breath (BB) time series were derived, allowing us to compute the PRQ time series as the ratio of instantaneous heart rate to instantaneous breathing rate. Linear indices of PRQ and RQA metrics were then calculated for the PRQ signals, enabling comparisons between groups (T2DM vs. control) and across tasks. Additionally, entropy-based mutual information (MI) between RRI and BB was assessed as a quantitative measure of cardiorespiratory coupling.

**Results:**

T2DM participants exhibited higher recurrence rates and prolonged recurrence time of the first type in the PRQ series, especially during paced breathing, suggesting a more rigid and less adaptive control mechanism. Although linear PRQ indices showed changes in some stage-dependent responses, they were less adept than RQA metrics at discerning subtle differences between groups. Furthermore, the complementary cardiorespiratory coupling assessment by MI revealed distinct compensatory patterns in T2DM during paced respiration and Valsalva.

**Conclusion:**

These findings indicate potential dysautonomia or partial autonomic dysregulation in individuals with T2DM, as reflected by altered cardiorespiratory dynamics and reduced adaptability.

## 1 Introduction

There is growing recognition in biomedicine that the human body functions as a complex and adaptive biological system where multiple organ subsystems dynamically interact through various pathways instead of depending on a hierarchical mode of control (Jayasinghe, 2012). Among the variety of physiological time series that reflect these interactions, heart rate variability (HRV) and, more recently, breathing rate variability (BRV) have been employed as non-invasive metrics to evaluate the autonomic control of cardiac and respiratory functions, respectively (Shaffer and Ginsberg, 2017; Soni and Muniyandi, 2019). Yet, standard linear measures of physiological signals can fail to capture the full intricacy of the underlying physiological processes, highlighting the advantage of nonlinear analytic methods in uncovering hidden regulatory mechanisms (Germán-Salló and Germán-Salló, 2016; Müller et al., 2017).

Type 2 diabetes mellitus (T2DM) can compromise the autonomic nervous system (ANS), leading to neurophysiological dysregulation that is evidenced by a decreased HRV, impaired baroreflex sensitivity, and altered cardiorespiratory coupling (Benichou et al., 2018; Kück et al., 2020; Da Silva et al., 2023). Growing evidence further indicates that T2DM is associated with a loss of systemic complexity (decomplexification), a phenomenon highlighted in Goldberger’s hypothesis, which posits that the intricate physiological complexity degrades with aging and during the course of chronic diseases (Goldberger et al., 2002). Along similar lines, Costa et al. reported a progressive reduction in the complexity of glycemic time series—emerging even before overt hyperglycemia—suggesting that these changes reflect the gradual breakdown of integrated control mechanisms that are central to the metabolic homeostasis (Costa et al., 2014).

Despite the well-documented impact of T2DM on autonomic cardiorespiratory activity, few studies have examined the Pulse-Respiration Quotient (PRQ)—the ratio of instantaneous heart rate to instantaneous breathing rate—in T2DM over shorter time frames. For instance, Bettermann et al. (2001) assessed 24-h data and found a reduction in cardiorespiratory coordination in diabetic patients (Bettermann et al., 2001). However, to the best of our knowledge, none study has investigated short-term PRQ in T2DM. This ratio is considered a marker of cardiorespiratory coupling, as it reflects the interplay between cardiac and respiratory rhythms (Scholkmann and Wolf, 2019). Conventional linear indices (e.g., the mean or standard deviation of PRQ) can detect differences between resting and orthostatic conditions in humans (Matić et al., 2022). Yet, nonlinear approaches, such as Recurrence Quantification Analysis (RQA), offer complementary insights into the structural changes in physiological time series (Webber and Zbilut, 1994). In fact, RQA has already been applied to HRV analysis, including studies on diabetic populations: for instance, Javorka et al. (2008) reported that young patients with type 1 diabetes mellitus exhibit a higher percentage of determinism in HRV signals compared with healthy controls (Javorka et al., 2008). Thus, conventional linear metrics of PRQ may not fully capture the cardiorespiratory changes associated with autonomic dysfunction in T2DM.

This study aims to investigate whether T2DM alters the short-term dynamics of PRQ time series through both linear and nonlinear analyses. Specifically, we compare diabetic and non-diabetic participants to assess potential changes in the RQA metrics of PRQ time series across different conditions and stages of a protocol that modulate the autonomic activity, which includes supine rest, orthostatic challenge, paced respiration, and the Valsalva maneuver. We hypothesize that individuals with T2DM will exhibit increased recurrence and reduced complexity in PRQ time series compared to healthy controls, with more pronounced differences during such tasks that challenge the cardiorespiratory regulation.

## 2 Methods

### 2.1 Description of participants

Recruitment and protocol testing took place between August 2023 and March 2024. This cross-sectional and exploratory study included a total of 38 participants: 18 in the control group and 20 with a confirmed diagnosis of T2DM. The sample size was determined based on prior methodological considerations established in the study protocol. Participants were then recruited using a non-probabilistic convenience sampling approach. All individuals were recruited at the “Nueva Oxtotitlán” Family Medicine Clinic, which is part of the Institute for Social Security and Services for State Workers (ISSSTE) in Toluca de Lerdo, State of Mexico, Mexico. Before enrollment, each participant signed a written informed consent. The study protocol was approved by the Research Ethics Committee of the School of Medicine at the Universidad Autónoma del Estado de México (CONBIOÉTICA-15-CEI-002-20210531, approval number 016_2023). The study adhered to the Mexican regulations for human research (NOM-012-SSA3-2012).

Participants were selected based on demographic and clinical parameters, with a focus on individuals aged 50 to 70. The control group was matched to have similar height and weight ranges as the T2DM group but without any known history of diabetes (Table 1). At the time of testing, fasting blood glucose levels in the control group were within normal ranges (93.6 ± 7.3 mg/dL). Meanwhile, the T2DM group consisted of individuals diagnosed with T2DM for at least 5 years, confirmed in a hospital setting by HbA1c or oral glucose tolerance tests. Although fasting glucose values for the T2DM group were not available at the time of testing, hospital physicians reported that their levels were stable or within normal ranges based on their clinical follow-up. Most T2DM participants were under pharmacological treatment, primarily with metformin. Additionally, four participants in the T2DM group had confirmed diabetic neuropathy, diagnosed through clinical evaluation. Diabetic neuropathy was further assessed using the standardized 10-point plantar sensitivity test with a 10 g-calibrated monofilament, considering the absence of sensitivity in more than four points as an indicator of neuropathy. Exclusion criteria included acute illness immediately before or during the test day, participation in intense physical activity within 24 h prior to the evaluation, or consumption of caffeine, energy drinks, or alcohol.

### 2.2 Experimental protocol and data acquisition

Following a brief acclimatization period in a quiet room, during which participants were in a supine position for approximately 5 min, each participant completed four experimental stages, during which cardiovascular and respiratory responses were continuously recorded via electrocardiogram (ECG) and respiratory recordings (RESP). In the first stage (S), participants rested in a supine position for 5 minutes. Then, they stood upright for 5 minutes in the second stage (O) for an orthostatic challenge assessment. In the third stage (R), while remaining in a supine position, they engaged in paced breathing or respiration at a rate of six breaths per minute for 3 minutes, guided by an auditory cue to maintain a synchronized respiratory rate at a 1:1 inspiration-to-expiration ratio. A trained professional supervised the participants to ensure the proper execution of the exercise. In the fourth stage (V), participants intermittently performed the Valsalva maneuver while seated. They were instructed to inhale for 5 s, hold their breath, and apply pressure on their lungs for 15 s with the mouth closed, then exhale for 5 s. This sequence was repeated for 2 min, leading to 5 to 6 repetitions of the maneuver. No participant reported dizziness or discomfort during any respiratory exercise. As the V stage had the shortest duration, analyses were restricted to the first 2 minutes of data in each stage to ensure consistency. Although the first 2 minutes of each stage may include transitional autonomic adjustments, we selected this interval to capture the immediate physiological responses to the experimental interventions. Given that the V stage was limited to 2 minutes, analyzing the same initial period across all stages ensured a consistent and directly comparable data set for evaluating rapid cardiovascular and respiratory dynamics.

**FIGURE 1:**
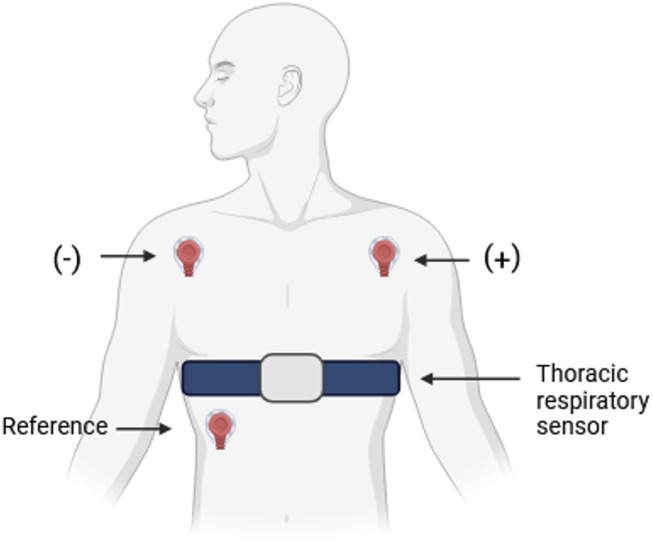
Schematic representation of the electrode configuration for electrocardiogram (ECG) acquisition and the positioning of the thoracic respiratory sensor. The ECG was recorded using a three-electrode bipolar lead system, with the negative electrode placed on the right infraclavicular region, the positive electrode on the left infraclavicular region, and the reference electrode positioned on the left lateral chest at the level of the fifth rib. The thoracic respiratory sensor belt was positioned around the chest to monitor respiratory activity (RESP).

Physiological data were captured at a sampling frequency of 128 Hz using a portable mobile amplifier, Mobi (TMSi Systems, Netherlands), transferred in real-time to a computer interface via Bluetooth 1.1. A three-electrode bipolar lead configuration was used for the ECG recordings, with the negative electrode placed in the right infraclavicular region, the positive electrode in the left infraclavicular region, and the ground electrode positioned on the left lateral chest at the level of the fifth rib. Additionally, a respiratory sensor belt was positioned to monitor respiration. Figure 1 illustrates the electrode placement for ECG acquisition and the positioning of the respiratory sensor belt.

### 2.3 Preprocessing and PRQ time series computation

The Pan–Tompkins algorithm was applied to the ECG recordings to detect R peaks, enabling the extraction of the R-to-R peak interval (RRI) series (Sedghamiz, 2020). In parallel, inspiratory peaks in the RESP signals were identified to form a breath-to-breath (BB) time series. Subsequently, both the RRI and BB time series underwent adaptive filtering to remove spurious heartbeats or breaths that were not representative of the true physiological signals (Wessel et al., 2000). Subsequently, each series was spline-interpolated at 4 Hz to ensure uniform time spacing and equidistant lengths (Zhao et al., 2019; Koppula et al., 2022), facilitating further analysis of the PRQ. This metric is used to evaluate potential alterations in both the linear and nonlinear aspects of cardiorespiratory dynamics (Matić et al., 2022). The PRQ is determined based on the RRI:BB ratio (m:1). It can be computed automatically by recording ECG data and respiration signals, extracting RRI and BB intervals, and directly calculating their ratio.

When RRI and BB time series are expressed as counts per minute, they correspond to instantaneous heart rate (HR) and instantaneous breathing rate (BR), respectively. These relationships are described by:

$$Heart\;rate\;\left(HR\right)=\frac{60}{RR}\;\left[\text{heartbeats}/\min\right]$$ (1)

$$Breathing\;rate\;\left(BR\right)=\frac{60}{BB}\;\left[\text{respirations}/\min\right]$$ (2)

$$PRQ=\frac{HR\;}{BR}$$ (3)

The PRQ analysis captures a distinct component of cardiorespiratory activity (Matić et al., 2022). In this study, we transformed the RRI and BB time series into instantaneous HR (Equation 1) and BR (Equation 2) to calculate the PRQ time series. The instantaneous PRQ was then determined by dividing the instantaneous HR by BR (Equation 3). According to Scholkmann and Wolf (Scholkmann and Wolf, 2019), it is essential to move beyond the standard method of calculating the PRQ by simply averaging the heart rate and breathing rate. Instead, an algorithm should be utilized to continuously compute heartbeat intervals for each respiratory cycle in real-time. In the present study, we used the interpolated and equidistant BR and HR time series to derive the PRQ time series over approximately 2-min segments. Here, the “functional PRQ” was examined. Notably, when the PRQ value stabilizes near 4—referred to as “PRQ normalization”—it signifies an optimal PRQ concerning cardiovascular function, highlighting the physiological significance of this state (Scholkmann et al., 2019).

Next, the PRQ time series were analyzed using both linear and nonlinear indices, including the mean PRQ (mPRQ) and the PRQ standard deviation (SDPRQ). These metrics have been shown to be sensitive to changes in the dynamic behavior of cardiorespiratory coupling, such as variations in body posture and breathing patterns. The mPRQ provides a summary measure of overall PRQ levels (Matić et al., 2022).

### 2.4 RQA analysis

To represent recurrence behavior within a time series or dataset, an *N* × *N* recurrence matrix is generated based on the criterion (Equation 4):

$$R_{i,j}=\Theta \left(\varepsilon_{i}-∥\vec{x_{i}}-\vec{x_{j}}∥\right),\vec{x_{i}},\vec{x_{j}}\in {R\;}^{m},i,j=1,\ldots ,N-\left(m-1\right)\tau$$ (4)

Where $R_{i,j}$ is the element at the *ith* row and *jth* column of the matrix, indicating whether the state at time *i* recurs at time *j*. The Heaviside step function Θ assigns a value of 1 if the distance between *x*_*i*_ and *x*_*j*​_ is smaller than the threshold *ε*_*i*_​, and 0 if it exceeds that threshold. The parameter *ε*_*i*_​ establishes the maximum distance allowed for two states to be considered recurrent and ∥⋅∥ (e.g., the Euclidean norm) measures the distance between the vectors in the reconstructed phase space.

The vectors *x*_*i*_ and *x*_*j*​_ lie in an *m*-dimensional phase space, ℝ^*m*^. The integer *m* is the embedding dimension, defining the number of delayed copies of the original time series used to rebuild the phase space. The embedding delay *τ* specifies the separation in time between these delayed copies, ensuring that the essential dynamics of the system are captured. A univariate time series *u*_*i*​_ is mapped onto this higher-dimensional space via (Equation 5):

$$\vec{x_{i}}=\left(u_{i},u_{i+\tau },\ldots ,u_{i+\left(m-1\right)\tau }\right)\text{ for }i=1,\ldots ,N-\left(m-1\right)\tau$$ (5)

Here, *i* and *j* range from 1 to $N-\left(m-1\right)\tau$ , where *N* is the length of the time series. For the current study, a one-dimensional series of PRQ was converted into an *m*-dimensional phase space using time-delay embedding. Each point in this reconstructed space represents the system’s state at a specific moment and is determined by the *m* coordinates corresponding to the embedding dimension.

**FIGURE 2:**
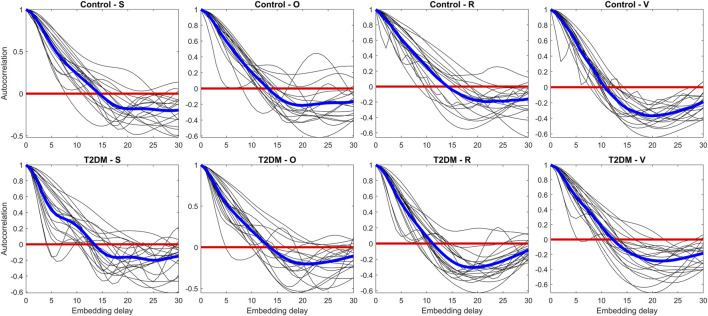
Assessment of the autocorrelation function for each Pulse-Respiration Quotient (PRQ) time series (thin lines) and averaged values per group (thick lines). The panels compare control participants (upper row) and individuals with type 2 diabetes mellitus (T2DM, lower row) across four experimental conditions: supine rest (S), orthostatic challenge (O), paced breathing (R), and the Valsalva maneuver (V). The autocorrelation function quantifies the temporal dependencies in the PRQ time series, providing insight into the underlying dynamics of autonomic regulation in response to different physiological challenges.

The embedding delay τ was chosen as the first zero-crossing of the mean autocorrelation function across all PRQ signals (Javorka et al., 2008; Calderón-Juárez et al., 2020). In Figure 2, thin lines depict the autocorrelation function for each individual recording, while thick lines represent the group averages for all the studied stages of Control and T2DM groups. The averaged autocorrelation function reached zero between lags 14 and 16, leading us to select τ = 14 for all subsequent analyses.

**FIGURE 3:**
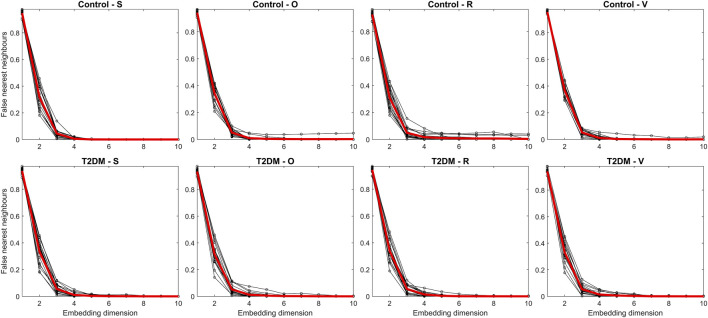
Assessment of the false nearest neighbors’ method for each Pulse-Respiration Quotient (PRQ) time series (thin lines) and averaged values per group (thick lines). The panels compare control participants (upper row) and individuals with type 2 diabetes mellitus (T2DM, lower row) across four experimental conditions: supine rest (S), orthostatic challenge (O), paced breathing (R), and the Valsalva maneuver (V). The false nearest neighbors’ method estimates the optimal embedding dimension for nonlinear analysis, helping to determine the minimal dimensionality required to reconstruct the system’s phase space and assess its underlying complexity.

Figure 3 illustrates the proportion of false nearest neighbors as a function of the embedding dimension for each recording and condition (thin lines) alongside the group-averaged values (thick lines). An overall rapid decrease in false neighbors was observed with increasing dimension. By an embedding dimension of 5, at least 50% of the recordings fulfilled the criterion of having fewer than 10% (i.e., 0.1) false nearest neighbors. Following the guideline by Abarbanel and Kennel (1993) (Abarbanel and Kennel, 1993), which suggests that an embedding dimension yielding fewer than 10% false neighbors is sufficient to recapture the system’s dynamics without significant projection errors, we selected *m* = 5. Distances between points in the reconstructed phase space were computed using the ‘fan’ option in the recurrence plots toolbox, which selects a fixed number of nearest neighbors. To ensure that each column of the recurrence plot has a local recurrence rate of 7%, we preselected the number of nearest neighbors corresponding to 7% recurrent points. The toolbox then automatically and dynamically determined the appropriate threshold, *ε*_*i*_, for each recording.

From these PRQ time series—acquired during the S, O, R, and V stages—several RQA metrics were calculated using the Cross Recurrence Plot Toolbox for MATLAB, Version 5.29 (R38) (Marwan et al., 2007; Marwan, 2024). The toolbox can be accessed at https://tocsy.pik-potsdam.de/CRPtoolbox/. Below is a concise overview of the RQA metrics used:

Recurrence rate (RR) represents the density of recurrence points within the recurrence plot (Marwan et al., 2007). The threshold *ε*_*i*_​ directly influences RR​, its corresponding formula is given in Equation 6:

$$RR=\frac{1}{N^{2}}\sum_{i,j=1}^{N}R_{i,j}$$ (6)

DET or determinism measures the fraction of recurrence points forming diagonal lines of at least (*l*_min_) points relative to the total number of recurrence points, thus reflecting the system’s predictability. In this study, *l*_min*=*_ *2* was chosen, which is a commonly used default value (Babaei et al., 2014). In Equation 7, *P(l)* represents the frequency distribution of these diagonal line lengths (Marwan et al., 2007).

$$\mathrm{DET}=\frac{\sum_{l=l_{\min}}^{N}l\cdot P\left(l\right)}{\sum_{l=1}^{N}l\cdot P\left(l\right)}$$ (7)

The *L* measure represents the mean prediction time over which trajectories in the phase space remain in proximity (Marwan et al., 2007). Formally, it is defined in Equation 8:

$$L=\frac{\sum_{{l=l}_{\min}}^{N}lP\left(l\right)}{\sum_{l=l_{\min}}^{N}P\left(l\right)}$$ (8)

LMAX is the length of the longest diagonal structure found in the recurrence plot, providing insights into the attractor’s stability (Marwan et al., 2007). Mathematically, it is defined in Equation 9:

$$LMAX=\max\left\{l|P\left(l\right)>0\right\}$$ (9)

Entropy (ENTR) evaluates the distribution of diagonal line lengths in the recurrence plot, specifically through the probability distribution *p(l)*, rather than providing a direct measure of system complexity. For uncorrected entropy, periodic dynamics can exhibit large ENTR values, chaotic dynamics can also have high ENTR, while random dynamics often yield relatively low ENTR. When border effect corrections are applied, the periodic dynamics tend to have lower entropy values (Kraemer and Marwan, 2019). Formally, it is formulated in Equation 10:

$$ENTR=-\sum_{l=l_{\min}}^{N}p\left(l\right)\log p\left(l\right)$$ (10)

Laminarity (LAM) calculates the fraction of recurrence points forming vertical lines in the recurrence plot, indicating that the system remains in a specific state for extended periods (Marwan et al., 2002). If *P*(*w*) represents the probability of finding a vertical line of length *w*, and *w*_min_ is the minimum length considered. In this study, *w*_min_ = 2 in accordance with standard practice (Marwan, 2024). LAM is expressed in Equation 11:

$$LAM=\frac{\sum_{{w=w}_{\min}}^{N}wP\left(w\right)}{\sum_{w=1}^{N}wP\left(w\right)}$$ (11)

Trapping time (TT) is the mean length of vertical lines in the recurrence plot, reflecting the stability of “trapped” states within the system (Marwan et al., 2002). It is expressed in Equation 12:

$$TT=\frac{\sum_{{w=w}_{\min}}^{N}wP\left(w\right)}{\sum_{{w=w}_{\min}}^{N}P\left(w\right)}$$ (12)

Recurrence time of the first type (T1) characterizes the average time interval between recurrent points along the *ith* column of a recurrence plot, interpreted as the mean duration for a state in the embedding space to reappear. Although other approaches to recurrence-time statistics exist (Ngamga et al., 2012), T1 offers a direct and intuitive measure suitable for our analysis. Formally, it is represented in Equation 13:

$$T1=\frac{1}{N}\sum_{i=1}^{N}T_{i}^{\left(1\right)}$$ (13)

Where $T_{i}^{\left(1\right)}$ represents the average of the minimum time difference between points in the neighborhood of a point *i* on the reconstructed trajectory.

Recurrence time of the second type (T2) measures the average time needed for a state to be revisited in the embedding space, excluding single time-unit intervals (Marwan et al., 2007). It is expressed in Equation 14:

$$T2=\frac{1}{N}\sum_{i=1}^{N}T_{i}^{\left(2\right)}$$ (14)

Where $T_{i}^{\left(2\right)}$ represents the average return time, defined as the minimum time difference between the recurrence points in the neighborhood of point *i* on the reconstructed trajectory, excluding all successive time points (Marwan et al., 2007).

We selected RQA because it is particularly effective for short and noisy physiological time series and is well-suited for signals produced by nonlinear systems (Chatain et al., 2021). Additionally, RQA has proven to be a versatile tool for investigating nonstationary data, aligning with the complex nature of the cardiorespiratory signals examined in this study.

### 2.5 Cardiorespiratory coupling assessment by mutal information (MI)

Mutual Information enables the assessment of information flow as a function of the time lag, τ. This method quantifies the information obtained from observations of one random variable in relation to another, capturing both linear and nonlinear dependencies between the two random variables, *X* and *Y*. In this study, the cross-mutual information (MI) function was used to quantify the cardiorespiratory coupling between two signals: $x\left(t\right)$ , representing the R-to-R peak interval (RRI) series, and $y\left(t\right)$ , representing the breath-to-breath (BB) time series.

MI is the nonlinear equivalent of the cross-correlation function and is based on the Shannon entropy, which measures the uncertainty of a random variable. The Shannon entropy of a time series $x\left(t\right)$ is calculated using its discrete probability distribution $p\left(x_{i}\left(t\right)\right)$ , resulting in $H_{x\left(t\right)}$ , as expressed in Equation 15:

$$H_{x\left(t\right)}=-\sum_{i=1}^{I}p\left(x_{i}\left(t\right)\right)\log_{2}\left(p\left(x_{i}\left(t\right)\right)\right)$$ (15)

where I represents the number of bins required to estimate the amplitude the histogram of $x\left(t\right)$ , which serves as an approximation of the signal’s probability distribution function (Hoyer et al., 2002). Similar formulas provide $H_{x\left(t\right)}$ and $H_{x\left(t\right)y\left(t+\tau \right)}$ . Here, $H_{x\left(t\right)}$ represents the entropy of $x\left(t\right)$ , and $H_{x\left(t\right)y\left(t+\tau \right)}$ is the joint entropy, which is calculated by summing over the bivariate probability distribution $p\left(x\left(t\right),y\left(t+\tau \right)\right)$ , as expressed in Equation 16:

$$H_{x\left(t\right),y\left(t+\tau \right)}=-\sum_{x\left(t\right)}\sum_{y\left(t+\tau \right)}p\left(x\left(t\right),y\left(t+\tau \right)\right)\log_{2}\left[p\left(x\left(t\right),y\left(t+\tau \right)\right)\right]$$ (16)

The MI between $x\left(t\right)$ and $y\left(t+\tau \right)$ is defined in Equation 17:

$$MI_{x\left(t\right)y\left(t+\tau \right)}=H_{x\left(t\right)}+H_{y\left(t\right)}-H_{x\left(t\right)y\left(t+\tau \right)}$$ (17)

where $H_{x\left(t\right)y\left(t+\tau \right)}$ is the joint entropy computed from the bivariate probability distribution of $x\left(t\right)$ and $y\left(t+\tau \right)$ . This joint entropy quantifies the shared uncertainty between two-time series, reflecting the amount of information one signal provides about the other $x\left(t\right)$ and $y\left(t+\tau \right)$ . A higher MI value indicates a stronger statistical dependence between $x\left(t\right)$ and $y\left(t+\tau \right)$ , while a lower MI value suggests weaker coupling (Pompe et al., 1998).

For this study, a function was programmed in MATLAB (The MathWorks, Inc., Natick, MA, United States, version R2023a) to calculate the mutual information between the RRI and BB time series. The maximum lag was set to *τ* = 30 based on an empirical evaluation of mutual information across different time delays, ensuring that the selected range effectively captured the interaction between both time series, as recommended by Pompe et al., 1998. Additionally, the Shannon entropy was estimated using a histogram approach with 10 bins.

### 2.6 Statistics

All variables were assessed for normality using the Kolmogorov–Smirnov test. For those meeting the normality assumption, we performed a two-way repeated-measures ANOVA with group (Control vs T2DM) as the between-subjects factor and time (S, O, R, and V) as the within-subjects factor. Post hoc comparisons were carried out using the Uncorrected Fisher’s LSD test, and for nonparametric data—including comparisons of MI—the Mann–Whitney test was used. Although multiple comparisons were made across the experimental phases (S, O, R, and V), given the exploratory nature of our study, no statistical corrections were applied. In exploratory studies, adjustment for multiple comparisons is not desirable for several reasons, as such corrections may increase the risk of type II errors by masking potentially meaningful trends. Instead, the analyses were performed without adjustment, with the understanding that additional dedicated studies are needed to confirm these results (Althouse, 2016). All statistical analyses were carried out using GraphPad Prism version 10.0.0 (GraphPad Software Inc., La Jolla, CA, United States), and a p-value less than 0.05 was considered statistically significant.

## 3 Results

Table 1 summarizes the clinical and demographic characteristics of the participants. Although most parameters showed no statistically significant differences between groups, the T2DM cohort exhibited elevated systolic and diastolic blood pressures compared to the Control group (109 ± 10/71 ± 7 mmHg vs 123 ± 15/79 ± 10 mmHg, p < 0.05).

**TABLE 1:** Clinical and demographic characteristics of the study population (mean ± SD).

Parameter	Control N = 18	T2DM N = 20
Age (years)	56 ± 6	56 ± 5
Weight (kg)	71.4 ± 10.3	79.8 ± 20.1
Height (m)	1.60 ± 0.07	1.61 ± 0.08
BMI (kg/m2)	27.6 ± 4.7	30.2 ± 5.6
Systolic blood pressure (mm/Hg)	109 ± 10^a^	123 ± 15
Diastolic blood pressure (mm/Hg)	71 ± 7^a^	79 ± 10
Gender (female, %)	61	65
Glucose (mg/dL)	93.6 ± 7.3	-
Time span since diagnosis of diabetes (years)	-	10 ± 6
Presence of diabetic neuropathy (n/N)	-	4/20

^a^p < 0.05 between Control vs T2DM.

**FIGURE 4:**
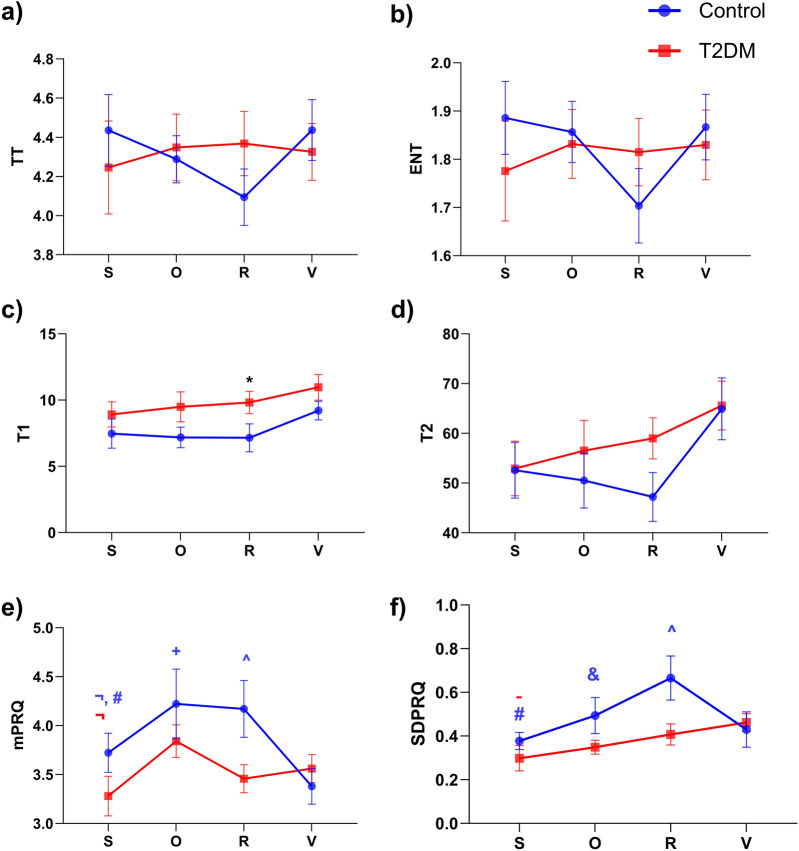
Comparison of recurrence quantification analysis (RQA) indices and linear indices for Pulse-Respiration Quotient (PRQ) time series across different experimental stages: supine rest (S), Orthostatic challenge (O), Paced respiration (R), and Valsalva maneuver (V) for control (blue) and diabetic (red) groups. Panels represent the following indices: **(a)** Trapping time (TT), **(b)** Entropy (ENT), **(c)** Recurrence time of first type (T1), **(d)** Recurrence time of second type (T2), **(e)** Mean of PRQ (mPRQ), and **(f)** Standard deviation of PRQ (SDPRQ). Data are expressed as mean ± SEM. Significant differences are indicated as follows: *p < 0.05 between Control vs T2DM; p < 0.05 for ^#^(S vs R), ^¬^(S vs O),^-^(S vs V), ^&^(O vs R), ^+^(O vs V), and ^(R vs V). The color of the symbol represents the group: blue for the control group and red for the diabetic group.

Regarding the mPRQ values (Figure 4e), a two-way repeated-measures ANOVA revealed a significant time × group interaction (F (3,108) = 2.939, p = 0.0365) and a main effect of time (F (3,108) = 5.970, p = 0.0008). Post hoc comparisons for Controls indicated that, from S (3.72 ± 0.85) to the orthostatic challenge (O: 4.22 ± 1.51), mPRQ increased significantly (p = 0.0283), also for S vs R (4.17 ± 1.23, p = 0.0485). Further comparisons showed differences between O and Valsalva (V: 3.38 ± 0.78, p = 0.0003) and between R and V (p = 0.0006). In T2DM, a significant increase in mPRQ occurred from S (3.28 ± 0.91) to O (3.84 ± 0.74, p = 0.0098), but no other stage-to-stage comparisons reached significance.

Analysis of the SDPRQ (Figure 4f) showed a significant main effect of time (F (3,108) = 4.441, p = 0.0055), but no overall group effect (F (1,36) = 3.666, p = 0.0635). Within the Control group, SDPRQ rose from S (0.38 ± 0.17) to R (0.66 ± 0.42, p = 0.0004) and showed additional within-group differences between O (0.49 ± 0.35) and R (p = 0.0331), as well as between R and V (0.43 ± 0.34, p = 0.0037). In T2DM, SDPRQ increased from S (0.30 ± 0.26) to V (0.46 ± 0.18, p = 0.0325), with no other significant changes between stages.

**FIGURE 5:**
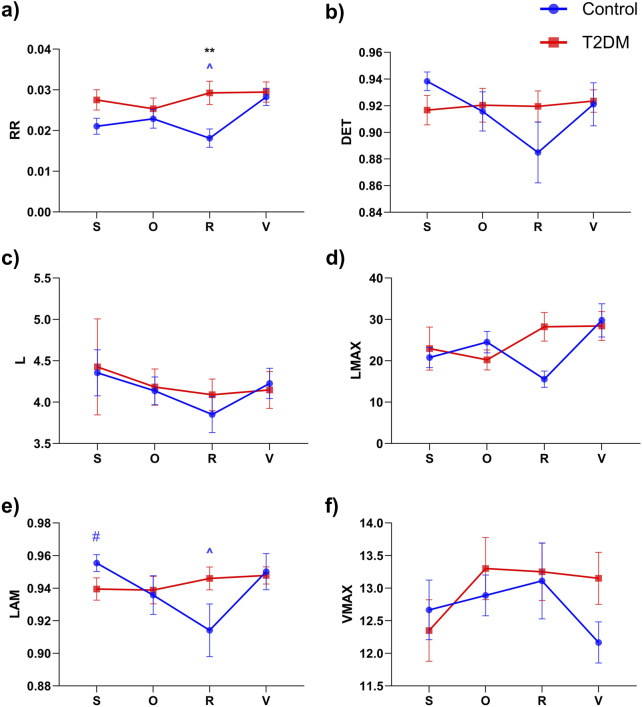
Comparison of recurrence quantification analysis (RQA) indices for Pulse-Respiration Quotient (PRQ) time series across different experimental stages: supine rest (S), Orthostatic challenge (O), Paced respiration (R), and Valsalva maneuver (V) for control (blue) and diabetic (red) groups. Panels represent the following RQA indices: **(a)** Recurrence Rate (RR), **(b)** Determinism (DET), **(c)** Mean diagonal line length (L), **(d)** Longest diagonal line (LMAX), **(e)** Laminarity (LAM), and **(f)** Maximum vertical line length (VMAX). Data are expressed as mean ± SEM. Significant differences are indicated as follows: **p < 0.01 between Control vs T2DM; p < 0.05 for ^#^(S vs R), and ^(R vs V). The symbol’s color represents the group: blue for the Control group and red for the T2DM group.

**FIGURE 6:**
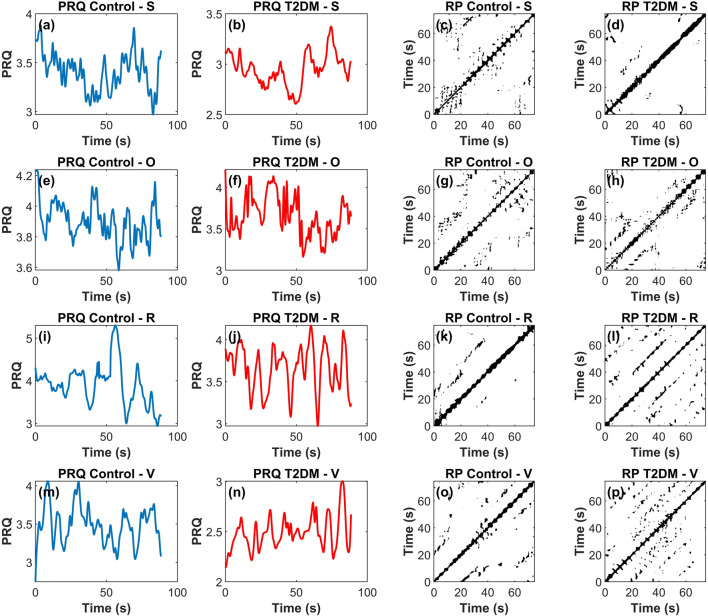
Representative example of Pulse-Respiration Quotient (PRQ) time series and corresponding recurrence plots across four experimental stages—supine rest (S), orthostatic challenge (O), paced respiration (R), and Valsalva maneuver (V)—for a control participant (blue) and a participant with type 2 diabetes mellitus (T2DM, red). In this 4 × 4 panel figure, each row corresponds to a stage (S, O, R, V) while the first and second columns display the PRQ time series for the control and T2DM groups, respectively, and the third and fourth columns show the corresponding recurrence plots. Panels **(a)**, **(b)**, **(e)**, **(f)**, **(i)**, **(j)**, **(m)**, and **(n)** correspond to PRQ time series, and panels **(c)**, **(d)**, **(g)**, **(h)**, **(k)**, **(l)**, **(o)**, and **(p)** to recurrence plots (RP).

Nonlinear analysis of PRQ time series via RQA (Figure 5) indicated a significant group effect for RR according to the two-way repeated-measures ANOVA (column factor: F (1,36) = 7.020, p = 0.0119). Post hoc tests revealed that during the R stage, RR was higher in the T2DM group than in Controls (p = 0.0015; 0.029 ± 0.13 vs 0.018 ± 0.10, Figure 5a). Within the Control group, RR also differed between R and V (p = 0.0028; 0.018 ± 0.10 vs 0.028 ± 0.009, Figure 5a). For the RQA index T1 (Figure 4c), the overall ANOVA showed a significant group effect (F (1,36) = 6.979, p = 0.0121). Although the pairwise comparison at the R stage yielded p = 0.0504, we still considered this a trend-level meaningful difference because of the numerical gap between the means of Controls (7.15 ± 4.48) and T2DM (9.81 ± 3.72). These findings are visually supported by Figure 6, which illustrates representative PRQ time series and recurrence plots for both groups. The T2DM group exhibits a regular or structured and repetitive PRQ signal (Figure 6j) compared to the presence of more fluctuations seen in the Control group (Figure 6i). Recurrence plots further highlight this contrast: while the Control group (Figure 6k) shows a more fragmented pattern, indicative of greater dynamical complexity, the T2DM group (Figure 6l) displays increased recurrence and longer diagonal structures, suggesting a more deterministic or rigid, and less adaptive cardiorespiratory interaction.

**FIGURE 7:**
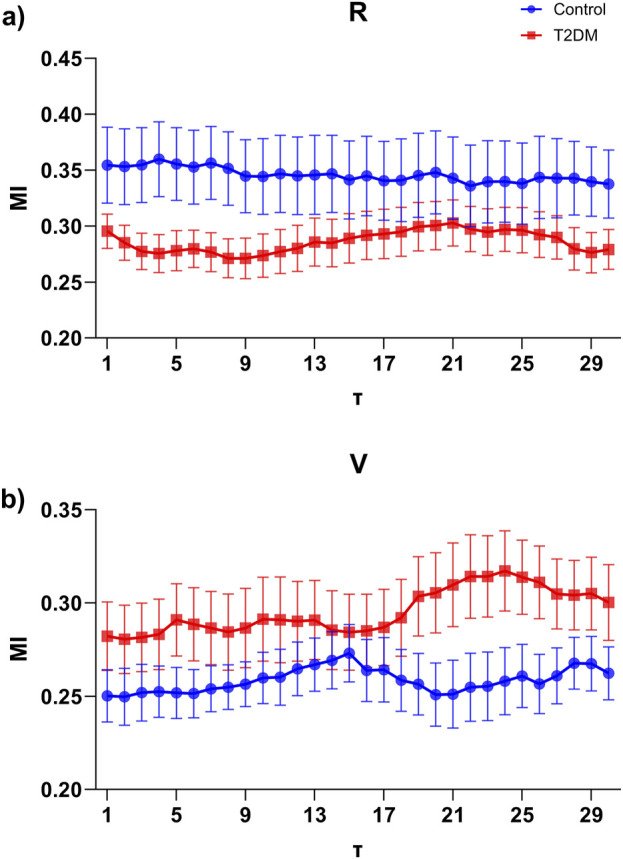
Mutual Information (MI) as a function of time delay (τ) ranging from 1 to 30, computed between interpolated R-to-R peak interval (RRI) and breath-to-breath (BB) signals in control (blue) and type 2 diabetes mellitus (T2DM, red) groups. **(a)** MI during the Paced respiration (R) stage, where the control group exhibits consistently higher MI values along delays compared to the T2DM group. **(b)** MI during the Valsalva maneuver (V) stage, showing an opposite trend, where the T2DM group presents higher MI values at larger time delays. Data are expressed as mean ± SEM.

The results shown in Figure 7 illustrate the MI across different time delays (τ) during the R and V stages for both groups. Although no statistically significant differences were observed at individual τ values, the overall MI behavior across the entire range was significantly different (p < 0.001). Specifically, during paced respiration (Figure 7a), the Control group consistently exhibited higher MI values compared to the T2DM group. Conversely, during Valsalva (Figure 7b), the T2DM group displayed a progressive increase in MI, surpassing the Control group at longer time delays. When averaging MI values across all τ within each condition, a statistically significant difference emerged between groups, indicating that the global dynamics of cardiorespiratory interaction differ between Control and T2DM participants.

We computed descriptive statistics for HRV and BB variability in both groups. In the control group, mean RRI values ranged from approximately 746–927 ms, with standard deviations between 134 and 215 ms, while mean BB values ranged from about 3,076 to 3,297 ms (SD ≈ 428–557 ms). In contrast, the T2DM group showed similar average RRI and BB values; however, during the O, R, and V stages, the T2DM participants exhibited lower RRI variability (with SDs of approximately 120, 150, and 152 ms, respectively) compared to controls (195, 215, and 191 ms, respectively). This reduced variability in RRI among T2DM subjects is consistent with our recurrence quantification analysis, which revealed higher recurrence rates and prolonged recurrence times in the PRQ series.

## 4 Discussion

The present findings underscore how nonlinear short-term analyses of the PRQ—particularly through RQA—can provide complementary insights of the cardiorespiratory function in individuals with T2DM. First, the RQA results demonstrated a significantly higher recurrence of the PRQ time series of T2DM individuals during the paced respiration stage compared to the non-diabetic group, possibly involving higher regularity in these time series. This result is in line with a previous work that suggests that chronic hyperglycemia and its associated autonomic dysfunction reduce the adaptive complexity of physiological signals (Javorka et al., 2008; Trunkvalterova et al., 2008). The increased T1 metric in T2DM further suggests that such participants exhibit prolonged intervals before returning to a similar state, reflecting a more deterministic and less adaptable cardiorespiratory control mechanisms.

Moura-Tonello et al., 2014 revealed that individuals with T2DM—even without cardiac autonomic neuropathy—can exhibit increased sympathetic modulation while being at rest, though their complexity indices (e.g., sample entropy) may remain comparable to healthy controls. Moreover, autonomic responses to postural changes appear largely preserved, suggesting that early-stage T2DM does not necessarily disrupt all aspects of the cardiac autonomic function. Although the results reported by Moura-Tonello et al. (2014) were obtained in the early stages of diabetes, the participants in our study have been living with the disease for approximately 10 years, which we believe may have influenced our findings. Furthermore, our results also align with evidence from Bassi et al. (2018), indicating that coexisting hypertension may exacerbate autonomic dysfunction in T2DM.

Second, it is noteworthy in particular that the significant changes in linear and nonlinear indices of the PRQ time series between stages—specifically from supine rest to paced respiration and from paced respiration to Valsalva—were observed exclusively in the Control group but not in T2DM. These findings may imply that healthy individuals could adjust more dynamically their cardiorespiratory rhythms under these varying physiological demands or challenges, whereas individuals with T2DM show a diminished capacity for such modulations. Reduced variability and diminished adaptive responses in diabetes may result from autonomic neuropathy or other diabetes-related impairments in cardiovascular control pathways (Jaiswal et al., 2013). For example, previous research has shown that patients with T2DM and microalbuminuria exhibit decreased HRV in response to respiratory and postural challenges, despite retaining a relatively normal blood pressure response (Chen et al., 2006). Our findings are consistent with this pattern, reflecting a partial preservation of certain autonomic responses (e.g., S vs O).

The regular physical activity in diabetic populations, which improves glycemic control and reduces blood pressure, has also been demonstrated to enhance HRV (Sridhar et al., 2010). While the present study did not directly assess exercise interventions, our results underscore the potential value of targeted training programs to restore or bolster cardiorespiratory adaptability, particularly in the light of the blunted responses we observed in stages like paced respiration and the Valsalva maneuver. In parallel, nerve conduction studies suggest that T2DM-associated neuropathy can simultaneously affect large motor and sensory fibers as well as smaller autonomic fibers, which could help explain the diminished capacity for moment-to-moment modulation of PRQ signals (Motataianu et al., 2021). Moreover, other research on type 1 diabetes has highlighted the possibility of early vagal impairment, manifesting in resting conditions despite still preserving responses to classic autonomic challenges (Castiglioni et al., 2022). Our data in T2DM similarly indicate that some traditional autonomic responses are preserved, but the underlying complexity and dynamic adaptive range are curtailed—a finding further supported by another study of T2DM women having good metabolic control, in which their HRV was consistently lower despite showing a relatively normal sympathetic-parasympathetic ratio (Robles-Cabrera et al., 2021).

Third, linear indices of the PRQ time series, including mPRQ and SDPRQ, reinforce their utility in detecting autonomic and postural changes. A mPRQ value near 4 has been consistently reported in healthy participants, suggesting optimal synchronization or phase-locking between cardiac and respiratory rates (Scholkmann and Wolf, 2019). In contrast, individuals with T2DM showed a less stable pattern around this physiological reference, as illustrated in Figure 4e. This observation suggests that diabetes may affect the delicate balance between cardiac and respiratory dynamics. It also highlights the relevance of linear PRQ analysis as a meaningful complement to nonlinear approaches like RQA in the assessment of autonomic function. Notably, while both mPRQ and SDPRQ were sensitive to orthostatic transitions and respiratory challenges, these indices did not reveal the differences between Control and T2DM that were by contrast evident by considering the RQA of PRQ time series. This suggests that RQA may detect subtler changes in the structure and complexity of PRQ time series, underlining the potential of this analysis for a more detailed clinical evaluation. Finally, the analysis of the MI between RRI and BB suggests that T2DM participants may exhibit distinct compensatory mechanisms during paced respiration and the Valsalva maneuver (Figure 7). Although no individual time delay showed statistically significant differences, the overall MI pattern of longer delays in Valsalva implies that T2DM could be associated with an impaired or incomplete autonomic response and an altered cardiorespiratory coupling (Da Silva et al., 2023). These observations raise the possibility that the dysautonomia, or partial autonomic dysregulation, is contributing to the reduced cardiorespiratory coupling in T2DM, especially during the more demanding respiratory challenges. Interestingly, previous studies using frequency-domain analyses, such as that by Rivera et al. (2016), show that healthy individuals maintain a well-defined respiratory peak during controlled breathing, indicating robust autonomic modulation. In contrast, T2DM tends to dampen this peak and reduce its frequency, hinting at a less flexible heart rate control. Together, these findings support the idea that diabetes may impair both vagal and sympathetic regulation of the heart, resulting in a diminished coupling between respiration and cardiac function—particularly evident during tasks that place higher demands on the autonomic nervous system (Rivera et al., 2016).

Together, these findings demonstrate how linear and nonlinear analyses of PRQ time series can be combined to obtain a more thorough insight of the ways in which T2DM affects the cardiorespiratory function. RQA and MI reveal more levels of complexity and structural organization, while the mean and standard-deviation metrics validate the PRQ’s sensitivity to reveal differences among typical physiological challenges. Aiming to help with individualized interventions and management strategies, an integrated approach using linear and nonlinear analyses may prove clinically relevant in detecting early or subclinical manifestations of the autonomic dysfunction in T2DM patients.

Previous research has successfully employed RQA to assess the coupling between cardiac and respiratory signals (Censi et al., 2002; 2015). In our study, the motivation for applying RQA to the PRQ stemmed from prior observations in COVID-19 survivors, particularly those with diabetes, where the linear and nonlinear analysis of PRQ showed trends approaching significance (Sánchez-Solís et al., 2023). Moreover, both linear and nonlinear analyses of PRQ in COVID-19 survivors during an induced relaxation test revealed statistically significant differences. These findings encouraged us to investigate RQA as a potentially sensitive tool for capturing subtle changes in cardiorespiratory coupling. While we acknowledge that alternative nonlinear metrics—such as detrended fluctuation analysis, multiscale entropy, symbolic analysis, or others—could also provide valuable insights into the complex dynamics of physiological signals. Although our current focus is on RQA due to its established applications and promising preliminary results, incorporating these additional methods in future studies could enrich the understanding of PRQ dynamics and offer a more comprehensive evaluation of cardiorespiratory interactions.

### 4.1 Limitations

Although we found significant differences in both linear and nonlinear metrics of the PRQ time series, our findings are limited in their depth and generalizability by a number of factors. First, given the exploratory nature of this study, the statistical power may be limited by the relatively small sample size (n = 38), which could account for the fact that we saw trends in the majority of RQA indices not reaching formal significance. Larger cohorts are necessary to confirm these preliminary results and ensure the repeatability of the observed patterns. Second, our cross-sectional design precludes causal inferences and fails to capture disease progression over time, which is especially relevant in T2DM, where participants may exhibit an evolving cardiac autonomic neuropathy (Williams et al., 2022). Additionally, our chosen sampling frequency of 128 Hz, while employed in other short-term signal-processing studies (Wang et al., 2018), is suboptimal for comprehensive ECG analysis compared to the recommended ≥1,000 Hz range. This limitation was recognized late in the study and may have constrained the depth of our nonlinear metrics, possibly affecting our results.

Although we instructed participants to avoid specific medications before testing, residual pharmacological effects cannot be entirely ruled out. Furthermore, the presence of comorbidities—particularly hypertension—could introduce confounding effects due to its strong association with T2DM and its known effects on cardiovascular autonomic control. Indeed, while our diabetic participants were generally under clinical management, their elevated blood pressure relative to controls may have influenced our results. Another consideration is the brief duration of our data segments, which primarily concentrated on swift responses (2-min recordings). Whereas such short-term analyses can reveal rapid autonomic adjustments, longer recordings (e.g., ≥5 min) may uncover additional aspects of the cardiorespiratory variability, potentially providing a more comprehensive assessment. We recognize that the brevity of our recordings aligns with some recent initiatives in ultra-short HRV research but may also reduce the reliability of certain metrics (Wehler et al., 2021). Longer data segments could better capture complex physiological patterns and enhance the robustness of both linear and nonlinear assessments. Future research should consider using higher sampling rates to enhance temporal resolution and ensure more robust estimations of cardiac autonomic function. Moreover, our T2DM group was not homogeneous with respect to the disease duration (ranging from 5 to 20 years) or sex distribution, both of which are known to impact the autonomic function (Koenig and Thayer, 2016; Dabelea et al., 2017).

A relevant study has specifically presented evidence of HRV as a biomarker for diabetic autonomic neuropathy in T2DM (Metelka et al., 2018), highlighting its usefulness as a convenient method for assessing varying degrees of autonomic dysfunction in diabetic populations. Although diabetic neuropathy could be considered a distinct subgroup or a more severe condition within individuals with T2DM, we chose to include the four participants diagnosed with diabetic neuropathy to maintain an adequate sample size representative of the broader T2DM population. Upon reviewing their data, we confirmed that these participants did not exhibit significant deviations from the remainder of the T2DM group regarding the primary outcomes of the present study. Nevertheless, we acknowledge this as a limitation, and we recommend that future research should separately consider groups of participants with T2DM, T2DM with diabetic neuropathy, and healthy controls, to better delineate the impact of diabetic neuropathy on autonomic function.

Finally, because most female participants were between 45 and 68 years of age—likely perimenopausal or postmenopausal—the influence of varying menstrual phases was considered minimal. Nevertheless, collecting more detailed information on menopausal status or hormone replacement therapy may be beneficial in future studies, particularly if a wider age range of female participants is included. Future studies should, therefore, increase sample size, stratify participants by sex, and consider grouping individuals by diabetes duration to better elucidate the interplay between T2DM severity and cardiorespiratory coupling. Furthermore, the use of MI to quantify cardiorespiratory coupling in the present study was based on prior research from our group, in which MI, alongside PRQ, was successfully applied to assess cardiorespiratory coupling in women with preeclampsia (Pichardo-Carmona et al., 2023). Although MI offers a simple way to quantify shared information between cardiovascular and respiratory signals, more advanced methods—such as Granger causality or transfer entropy—may provide deeper insights into the causal structure of the interaction and are planned for future analyses.

## 5 Conclusion

Our findings suggest that the RQA of short-term PRQ time series may offer valuable and complementary information on cardiorespiratory dynamics in T2DM. During paced respiration, T2DM participants exhibited significantly higher recurrence and T1 values compared to controls, indicating more rigid and less adaptive cardiorespiratory dynamics. Furthermore, while control participants showed several significant PRQ differences across stages of the experimental protocol, this was not the case for T2DM participants. Thereby suggesting a reduced capacity for cardiorespiratory adjustments in diabetes. Linear indices of the PRQ time series proved useful for detecting posture-related and autonomic changes—particularly once considering the fact that an mPRQ value close to 4 is a recognized norm in healthy individuals. Yet, such indices did not capture certain alterations revealed by RQA. This underscores the potential clinical benefit of combining linear and nonlinear assessments. Lastly, the MI analysis of RRI and BB time series suggests that T2DM could involve a lower cardiorespiratory coupling compared to controls during paced respiration accompanied by incomplete compensatory responses during more demanding tasks, such as those introduced by the Valsalva maneuver. This pattern likely reflects the effects of dysautonomia or partial autonomic dysregulation, which compromise the optimal cardiorespiratory coupling in diabetes.

## Data Availability

The raw data supporting the conclusions of this article will be made available by the authors, without undue reservation.
